# Strengthening immunization programs through innovative sub-national public-private partnerships in selected provinces in the Democratic Republic of the Congo

**DOI:** 10.1016/j.vaccine.2023.11.029

**Published:** 2023-12-12

**Authors:** Patrick Banza Mpiongo, Jerry Kibanza, Francis Kambol Yav, Didier Nyombo, Lucie Mwepu, Djogo Basame, Raoul Mpoyi, Collard Madika, Trad Hatton, Eric Mafuta, Oriane Gascon, Kevin Tschirhart, Freddy Nkosi, Paul Lusamba, Sydney Merritt, Julio Mwenda, Sylvia Tangney, Nicole A. Hoff, Dalau Nkamba Mukadi, Anne W. Rimoin, Didine Kaba, Amine El Mourid, Kamel Senouci, Guillaume Ngoie Mwamba, Elisabeth Mukamba Musenga, Aimé Cikomola

**Affiliations:** a Provincial Division of Health, Ministry of Health, Haut Lomami, Democratic Republic of the Congo; b Provincial Division of Health, Ministry of Health, Tanganyika, Democratic Republic of the Congo; c Provincial Division of Health, Ministry of Health, Lualaba, Democratic Republic of the Congo; d McKing Consulting, Kinshasa, Democratic Republic of the Congo; e PATH, Kinshasa, Democratic Republic of the Congo; f Kinshasa School of Public Health, Kinshasa, Democratic Republic of the Congo; g Independent Consultant; h GRID3, Columbia University, NY, USA; i VillageReach RDC, Kinshasa, Democratic Republic of the Congo; j Department of Epidemiology, Fielding School of Public Health, University of California, Los Angeles, CA, USA; k Bill and Melinda Gates Foundation, Seattle, WA, USA; l University of Geneva, Geneva, Switzerland; m Expanded Programme for Immunization, Minister of Health, Kinshasa, Democratic Republic of the Congo

**Keywords:** Routine immunization, Memorandums of understanding, Provincial autonomy, Vaccine, DRC

## Abstract

**Background:**

Low immunization coverage rates in the Democratic Republic of Congo (DRC) have been reflective of challenges with vaccine access, support and delivery in the country. Motivated by measles and vaccine-derived polio virus (VDPV) outbreaks in 2016–17 and low vaccination rates, the provinces of Haut Lomami and Tanganyika were identified as pilot locations for an innovative approach focused on establishing a consortium of partners supporting local government. This approach was formalized through Memorandums of Understanding (MoUs) between the Bill and Melinda Gates Foundation and Provincial governments in 2018. A third province, Lualaba, established an MoU in 2021.

**MoU implementation:**

These MoUs were 5-year partnerships designed to aid provinces in meeting four key objectives: 80 % immunization coverage, management/elimination of polio/cVDPV outbreaks, improvement of vaccine accessibility, and transfer of immunization service management to provincial leadership.

**Outcomes:**

During the MoU period, Haut-Lomami saw an increase in full immunization coverage, from 35.7 % (MICS 2018) to 88.9 % (VCS 2021–22), the highest in country. A sharp drop in percentage of zero-dose children was observed in the 3 provinces, confirming improved access to immunization services. Tanganyika saw initial improvement in full immunization coverage, followed by a drop in the VCS 2021–22 due to COVID-19 and healthcare worker strikes. Coverage improved in Tanganyika in the 2023 VCS. The 3 provinces increased their financial contributions to routine immunization and are now the top contributing provinces. While no cVDPV cases were recorded in 2020 and 2021, cVDPV1 and cVDPV2 outbreaks are afflicting the 3 provinces since 2022.

**Conclusions:**

Ultimately, the provincial MoUs were successful in bolstering provincial autonomy and capacity building with the biggest success being a drop in zero-dose children. While not all objectives have been met, the MoU approach served as an innovative program for key aspects of strengthening routine immunization in the DRC.

## Highlights

• MoU approach was an innovative program linking public-private collaborations.
• Sub-national government engagement for immunization program ownership.
• Potential for replication in other provinces and countries.

## 1 Introduction

In 2016 and 2017, the Democratic Republic of the Congo (DRC) suffered from a country-wide measles outbreak and circulating vaccine-derived polio outbreaks (cVDPV2) in three provinces: Haut Lomami, Tanganyika and Maniema [1]. Nationally representative surveys estimated that Haut Lomami and Tanganyika had less than 50 % full immunization coverage; Multiple Indicator Cluster Studies (MICS) in 2017–18 estimated 35.7 % full immunization coverage in Haut Lomami and 21.2 % full immunization coverage in Tanganyika, with more than 10 % zero-dose children, and child mortality greater than 40 per 1000 live births [2]. By mid-2018, both Tanganyika and Haut Lomami continued to report cases of cVDPV2, despite multiple rounds of supplemental immunization activities (SIAs) with the monovalent oral polio vaccine (mOPV) [3]. Persistent outbreaks and low vaccination coverage highlighted the need to improve the quality of the outbreak responses and bolster the existing vaccine-delivery system [4], [5]. In response, the Bill and Melinda Gates Foundation (BMGF) and both provincial governments launched a subnational-focused, innovative program inspired by the Nigerian state-level Memorandums of Understanding (MoU) approach, to raise immunization coverage levels [6].

Beginning in 2012, Nigeria implemented a MoU with BMGF, the Aliko Dangote Foundation, and six state governments [6]. The MoU goal was to strengthen the Routine Immunization (RI) system in Nigeria by addressing systemic challenges such as failed cold chains, vaccine distribution issues, insufficient health care worker supervision, data quality issues, and inadequate community engagement [6], [7]. A critical component of the MoU was the establishment of a basket fund where partners and the state government would fund immunization activities [6]. Over time, the financial contributions by states into the basket funds would gradually increase and ultimately cover all immunization costs. These MoUs were a unique approach to addressing sub-national-level challenges in RI and empowering states to self-finance and manage their immunization programs [7].

DRC adopted a decentralization law in 2007 that made health a provincial competency and priority [8]. In 2016, decentralization went into effect with the division of the country from 11 provinces to 26 provinces, with new institutions and administrative positions [9]. Despite the law, in practice, RI continued to be almost entirely managed by the national EPI program. Most of the RI specific funding was channeled through the national level.

To improve immunization rates in the DRC, the provincial governments of Tanganyika and Haut-Lomami signed similar MoUs with BMGF in October 2018. These MoUs incorporated a number of lessons learned from the previous Nigeria MoUs including the multi-stage process of initiation, implementation, and eventual transition of RI responsibilities to provincial, or state, governments. Furthermore, experiences in Nigeria shaped the establishment of basket funds in DRC[10]. The Haut Lomami and Tanganyika MoU launch coincided with the launch of the Emergency Plan for Revitalization of the Routine Immunization System, also called the Mashako Plan (MP) in the DRC [11]. This plan was designed to engage the subnational governments to improve immunization coverage in 9 pilot provinces, including Tanganyika and Haut-Lomami [11]. These provinces were specifically chosen due to their continued vaccine preventable disease (VPD) outbreaks and low immunization rates. The Haut Lomami and Tanganyika MoUs were signed by provincial governors and were designed to last for 51 months from October 2018 to December 2022 [12]. In July 2021, BMGF signed an additional MoU with the Lualaba province that would last until December 2023 [13].

In a global context, these MoUs fall under the broader goals of the World Health Assembly’s Immunization Agenda 2030 (IA2030). While signed and implemented before the declaration of IA2030 in 2020, the priorities of the MoUs align with the Assembly’s declaration to save over 50 million lives through vaccination [14]. IA2030 focuses on themes of ownership and accountability, monitoring and evaluation, and communication at both the national and sub-national levels—all central concepts of the MoUs in Haut Lomami and Tanganyika.

This article describes the design, implementation, and impact of the MoU program for immunization in the provinces that implemented this model. Implementation of the MoUs was expected both to improve vaccine coverage rates, and, at a more political and financial level, to increase subnational commitment and involvement in RI activities. Beyond Haut Lomami, Tanganyika and Lualaba, these MoUs provide a framework for improving sustainability and impact of public health initiatives.

## 2 MoU implementation

The signed MoUs with Haut Lomami, Tanganyika and Lualaba had four primary objectives. The first objective was to optimize immunization delivery to achieve at least 80 % immunization coverage (defined as receiving the third dose of pentavalent vaccine, or Penta3). Additionally, the MoUs aimed to stop the spread of VDPVs, keep provinces free from Polio and other VPDs, as well as improve immunization equity and reduce child morbidity and mortality. Finally, these agreements were set to transfer full responsibility of the immunization program management entirely to the provinces by December 2022.

The core philosophy of the MoUs was to empower provinces to self-manage their immunization programs and increase subnational decision making. The governor of each province was the executive sponsor of the approach, while the Provincial Health Director (Chef de Division Provinciale de Santé (DPS)) was designated as the leader of MoU implementation. In order to achieve this goal, these memorandums were rooted in four key principles: (1) provincial empowerment, (2) increased provincial financial contributions and financial management of the program, (3) accountability for reaching the service delivery targets set by the national Mashako Plan objectives, and (4) transfer of expertise from the technical assistance to the Provincial Division of Health.

### 2.1 MoU Principle 1 – provincial empowerment

The MoU set three levels of coordination in the executive branch to ensure accountability in each province. The provincial governor would ensure political commitment at the highest level was ultimately accountable for the implementation of the MoU, including the disbursement of funds. They would hold semi-annual meetings to evaluate immunization coverage improvements, discuss strategic decisions, and fund disbursements during the provincial health steering committees. Additionally, health zone managers received feedback from the governor based on their performance (letters of warning or commendation to the staff). The provincial ministers of health chaired monthly assessments and reviews of Mashako Plan indicators, vaccine coverage data, and provincial implementation. Finally, the provincial division of health held weekly Mashako Plan technical committee meetings to discuss day-to-day aspects of the routine immunization program.

The provincial parliament assemblies in charge of the legislative process requested regular updates of the MoU implementation. Provincial assemblies consist of elected representatives and are the legislative body in each of the 26 provinces of the DRC. Under the MoU, the assemblies of Haut Lomami and Tanganyika voted to include not only funding for immunization in the provincial budgets, but also to coordinate and review budget executions. Elected representatives were involved, and used their parliamentary recess to monitor the implementation of immunization activities within their constituencies.

To cement political motivation, under the Mashako Plan, the President of the country held bi-annual Presidential Forums in the capital city, Kinshasa, to assess indicators by province with all provincial governors in attendance. While the Forums and the Mashako Plan increased political will, set goals, and developed a standardized monitoring system for improved routine immunization, the MoUs provided an approach to implement more sub-national ownership to ultimately achieve these targets.

### 2.2 MoU Principle 2 – provincial financial independence

Prior to the MoU implementation, funding for RI operations activities was provided almost exclusively by donors (Gavi, UNICEF, USAID, World Bank, etc.). RI budgets were set at the national level with minimal provincial involvement, and disbursements were often delayed due to processing time, delays or issues with justifying funds from the previous quarter. In addition, provinces were usually expected to justify all funds and return any unused funds each quarter.

**Fig. 1:**
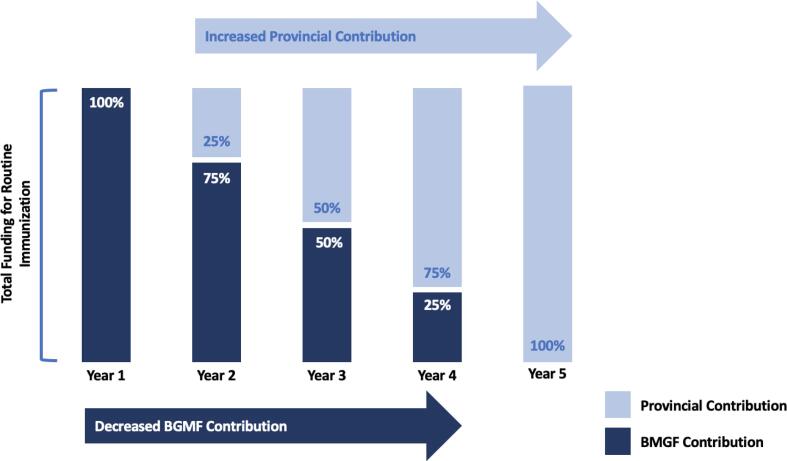
Building Financial Autonomy: Distribution of RI funding contributions over the course of the provincial Memorandums of Understanding.

To improve financial sustainability, pooled basket funds were established in each signatory province with both BMGF and provincial governments contributing to immunization activities. In these RI-specific basket funds, funding from BMGF was expected to decrease gradually as province contribution was expected to increase reciprocally (Fig. 1 **and** Table 1). This arrangement allowed for a large initial investment from an international donor with progressively increasing government support. An annual budget plan was developed, jointly approved, and regularly reviewed by both public provincial authorities and private partners, in accordance with national guidelines and directives. Funds were available at the beginning of the year and the balance of unused funds was available to be reprogrammed for the following year, to ensure continuity of activities. In addition to funding by BMGF, primary oversight of fund utilization and spending was conducted by the primary fiduciary agent of basket funds, PATH.

**Table 1:** Expected contributions for each province and BMGF under the MoU agreement.

**Haut-Lomami**	**2019**	**2020**	**2021**	**2022**	**2023**
Initial expected provincial contributions (k USD)	0	250	500	750	1000
Adjusted expected provincial contributions (k USD)	0	250	125	300	400
BMGF Expected contribution (k USD)	1000	750	500	250	0
**Tanganyika**	**2019**	**2020**	**2021**	**2022**	**2023**
Initial expected provincial contributions (k USD)	0	250	500	750	1000
Adjusted expected provincial contributions (k USD)	0	250	120	200	250
BMGF Expected contribution (k USD)	1000	750	500	250	0
**Lualaba**			**2021**	**2022**	**2023**
Initial expected provincial contributions (k USD)	–	–	300	400	500
BMGF Expected contribution (k USD)	–	–	400	200	150

Financial management was conducted by a specified core group as dictated by the MoU. For example, in Haut Lomami, the core group included the provincial EPI manager (Médecin Chef d’Antenne) as the acting president, a McKing provincial consultant representing the donors as vice-president, the financial advisor to the governor, the provincial director of the budget, the director of the provincial Ministry of Health (MoH) cabinet, the provincial PROSANI director (USAID funded health program in DRC) and a PATH technical officer. In each province, core group responsibilities included review, validation and monitoring of the annual workplan, budget development, and validation of funding requests in accordance with the workplan.

**Fig. 2:**
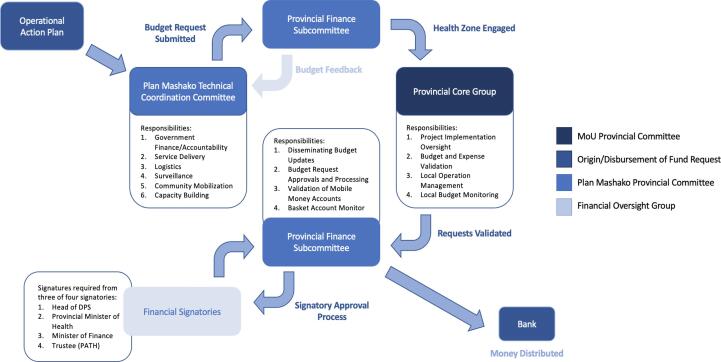
Financial approval process and affiliate groups as detailed in the Memorandum of Understanding.

Each Mashako Plan thematic group would send a request to the financial subcommittee, which would transfer it to the Core group for review. If both the finance subcommittee and the Core group approved the budget of the activity, the request would be validated (Fig. 2). Fund disbursement required signatures from three of the four following offices: Provincial Health Director, Minister of Health, Minister of Finance, and the primary trustee, PATH. PATH approvals were required for the first three years of basket fund access and use.

Prior to the implementation of the MoU, daily financial management of RI implementation was plagued by many challenges including disbursement tracking and justification. In order to overcome these challenges, the MoU proposed the use of financial and accounting management software, as well as training of the personnel of the division to use it. The provinces adopted a “Zero-Cash Policy” for disbursement from the basket fund, which stipulated that all funds would be distributed electronically via bank transfer and mobile money and avoid cash-disbursement to reduce unjustified or misuse of funds. The MoU provided phones with Mpesa accounts (a local money transfer program in collaboration with the Vodacom phone network) to users in the health zones and health facilities to improve monitoring of activities, tracking of funds, and ensure direct transfer of funds.

While the Core group and provincial finance committees had monetary oversight of budget development and program spending, the MoU also called for external audits of basket funds and management mechanisms to be conducted by an independent auditing company, in addition to ongoing internal audits. The external audit was included to increase confidence and accountability in the management processes. Basket funds, zero-cash policy, updated accounting system, and the manual of procedures were essential to the co-management of funds and increased provincial financial autonomy.

### 2.3 MoU Principle 3 - provincial accountability to achieve Mashako plan objectives and interventions to improve service delivery

Each province set up a Mashako Plan technical committee with thematic areas: governance (finance and accountability), service delivery, logistics, surveillance, M&E (monitoring and evaluation), community mobilization and advocacy, and capacity building. Through coordination between the national level Mashako Plan technical committee, provincial leadership, the financial subcommittee and MoU-designed Core group, MoU adherence was maintained and assessed regularly. The Mashako Plan also designed process indicators to track progress in the main components of RI. These process indicators were collected through monthly supervision of health facilities by a custom-built mobile supervision application. Additionally, vaccine coverage surveys at the health zone level were organized regularly to independently verify immunization outcomes. These output and technical measure outcomes provided a standardized template to compare performance between provinces implementing the Mashako Plan.

### 2.4 MoU Principle 4 - systematic reduction of external donor-supported technical assistance

Historically, several external organizations provided technical assistance to the RI program, each with different technical expertise: logistics, monitoring and evaluation, surveillance, service delivery, social mobilization, training, and overall coordination. While these organizations helped implementation of the programs, local coordination was poor between partners. In order to increase provincial autonomy and program ownership, the fourth MoU principle was the gradual reduction of external assistance provided to each participating province. Technical assistance provided support for provincial staff to implement critical interventions to address weak or non-functional systems in the immunization program.

This assistance was coordinated by PATH and included the establishment of a technical consortium which included: McKing consultants (acting as the overall advisor to the province), VillageReach, Caritas, Soins de Santé Primaires en Milieu Rural (SANRU), International Medical Corps (IMC), University of California, Los Angeles (UCLA), Acasus, Geo-Referenced Infrastructure and Demographic Data for Development (GRID3), New Horizons/Global health Labs (GHL), Bull City Learning, and other subcontractors. This consortium developed transition plans to reduce and eventually withdraw support from Haut Lomami and Tanganyika over the course of the MoU period.

The barriers to last-mile vaccine distribution in the DRC was the availability and functionality of cold chain, as well as adequate management systems to deliver vaccines. Under the consortium, VillageReach was the logistics technical advisor and supported the implementation of an improved vaccine-delivery system to the last mile: the New Generation Supply Chain (NAGA) system. This approach to vaccine delivery consisted in directly delivering vaccines to each health center (push) as opposed to traditional systems of health centers individually collecting their vaccine supplies from the district (pull). In addition, the MoU provided funding for the installation of cold chains in each health area (health zones are subdivided in health areas in DRC).

In terms of service delivery, MoU implementation led to changes in the immunization service delivery process itself. Three major changes were introduced: automated microplanning with satellite technology through GRID3, geolocated immunization session-tracking (digitized through use of a SMS-based mobile application developed by Acasus), and use of mobile money to make direct payments to healthcare workers (HCWs) performing immunization activities. Monitoring and evaluation were also supported by Kinshasa School of Public Health (KSPH) annual vaccine coverage surveys (VCS), real-time monthly monitoring with the EPI Supervision mobile application, and UCLA/KPSH indicator evaluations. From a community engagement perspective, Caritas, SANRU, and IMC generally worked to improve vaccine demand and implement community-based surveillance. Through increased communication of immunization schedules and identification of “zero-dose” and under-immunized children, these partners assisted local leaders in promoting vaccination campaigns and garnering vaccine demand.

**Fig. 3:**
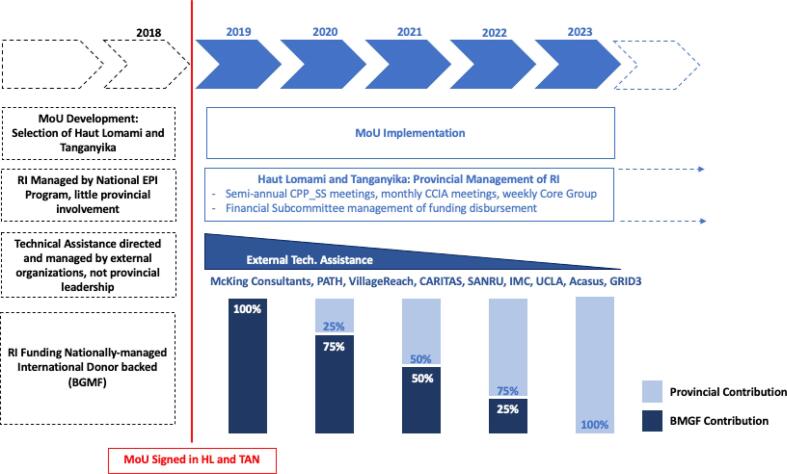
Timeline of MoU implementation by MoU principle.

At the beginning of the MoU period, the technical assistance consortium played a major role in capacity building, training of provincial leadership, and overall support. However, this support was intentionally designed to decrease overtime with external partners shifting to advisory roles as provinces took ownership of their RI system. Ultimately, following the conclusion of the MoU period, provincial leadership will manage all RI technical aspects independently. All implementation aspects of the MoU are summarized by principle in Fig. 3.

## 3 Outcomes – four objectives of MoU implementation

### 3.1 MoU Objective 1 – optimizing immunization coverage

Implementation of the MoU was designed with the primary objective to increase full immunization coverage to at least 80 %, with the expectation that the number of zero-dose children would similarly decrease. Full immunization coverage increased in Haut Lomami from 35.7 % in the 2018 MICS to 88.9 % in the 2021–2022 VCS (Table 2), over the course of the MoU implementation. By 2022, Haut-Lomami had surpassed the initial objective, and had the highest vaccination coverage rates nationwide. Additionally, by the 2021–2022 VCS, Haut Lomami only reported 2.4 % of zero-dose children compared to 11.0 % overall in the 9 initial Mashako Plan Provinces. These results were confirmed in the VCS 2023 survey, with 88.9 % of children fully immunized and 2.6 % zero-dose children.

**Table 2:** Vaccine coverage by province from various representative surveys 2013–2022.

**Haut Lomami**					
**Vaccine**	**MICS 2018 (n = 95)**	**VCS 2019 (n = 1,039)**	**VCS 2020 (n = 1,743)**	**VCS 2021**–**22 (n = 1,721)**	**VCS 2023 (n = 1,783)**
Full Immunization	35.7 %	39.0 %	69.4 %	88.9 %	88.9 %
Penta 1	63.4 %	59.7 %	89.8 %	97.6 %	97.4 %
Penta 3	50.4 %	50.4 %	77.9 %	94.5 %	94.8 %
Measles	54.2 %	50.2 %	84.5 %	93.2 %	93.0 %
Zero-Dose*	36.6 %	40.3 %	10.2 %	2.4 %	2.6 %
**Tanganyika**					
**Vaccine**	**MICS 2018 (n = 108)**	**VCS 2019 (n = 862)**	**VCS2020 (n = 1,475)**	**VCS 2021**–**22 (n = 1,116)**	**VCS 2023 (n = 1,051)**
Full Immunization	21.2 %	52.1 %	46.4 %	13.9 %	27.6 %
Penta 1	43.3 %	67.7 %	83.6 %	81.0 %	87.5 %
Penta 3	25.2 %	60.8 %	63.2 %	32.0 %	50.6 %
Measles	35.8 %	64.4 %	67.1 %	20.7 %	32.5 %
Zero-Dose*	56.7 %	32.3 %	16.4 %	19.0 %	12.5 %
**Lualaba**					
**Vaccine**	**MICS 2018 (n = 148)**	**VCS 2019 (n = 0)**	**VCS 2020 (n = 0)**	**VCS 2021**–**22 (n = 1,489)**	**VCS 2023 (n = 1,334)**
Full Immunization	21.1 %	–	–	36.7 %	44.2 %
Penta 1	51.6 %	–	–	81.7 %	79.8 %
Penta 3	34.7 %	–	–	58.6 %	59.9 %
Measles	42.2 %	–	–	57.6 %	51.4 %
Zero-Dose*	48.4 %	–	–	18.3 %	20.2 %
**9 Original Plan Mashako Provinces**					
**Vaccine**	**MICS 2018 (n = 1,736)**	**VCS 2019 (n = 0)**	**VCS 2020 (n = 18,592)**	**VCS 2021**–**22 (n = 18,777)**	**VCS 2023 (n = 16,777)**
Full Immunization	31.7 %	–	56.5 %	50.3 %	53.4 %
Penta 1	75.2 %	–	86.0 %	84.7 %	84.5 %
Penta 3	52.6 %	–	71.2 %	65.3 %	67.3 %
Measles	66.3 %	–	73.4 %	63.7 %	63.5 %
Zero-Dose*	24.8 %	–	14.0 %	15.3 %	15.5 %
**Country (26 provinces)**					
**Vaccine**	**MICS 2018 (n = 4287)**	**VCS 2019 (n = 0)**	VCS 2020 (n = 46,093)**	**VCS 2021**–**22 (n = 88,592)**	**VCS 2023 (n = 47,880)**
Full Immunization	35.0 %	–	52.5 %	41.5 %	45.3 %
Penta 1	65.8 %	–	83.2 %	80.9 %	81.2 %
Penta 3	47.6 %	–	67.6 %	60.3 %	61.3 %
Measles	57.2 %	–	68.5 %	55.9 %	56.1 %
Zero-Dose*	34.2 %	–	16.8 %	19.1 %	18.8 %

* ”Zero-dose” is defined via the Gavi definition of: 100 %-Penta1.

** Data from 18 provinces (Haut Katanga, Haut Lomami, Ituri, Kasai, Kasai Central, Kasai Oriental, Kinshasa, Kongo Central, Kango, Kwilu, Lomami, Maniema, Mongala, Sankuru, Sud Kivu, Tanganyika, Tshopo, Tshuapa).

In Tanganyika, after an initial increase was observed in the 2020 VCS (46.4 %) compared to the MICS 2018 (21.2 %), full immunization coverage dropped in the 2021–22 VCS (13.9 %). Drops in coverage in Tanganyika Province have been attributed to 3 major events: (1) interethnic conflict that started in 2016 and culminated in 2020 causing displacement of 18 % of the population; (2) in 2020, COVID-19 related rumors caused fear by the population of the vaccine being tested on children reducing demand for immunization services, which caused a drop in immunization session attendance; and (3) in 2021, large scale strikes by HCWs resulted in closures of health facilities for almost 6 months resulting in the interruption of delivery of routine immunization services. A year later, full immunization coverage almost doubled to 27.9 % according to the 2023 VCS. There was also a reduction in zero-dose children in the province between the 2018 MICS, 2021–2022 VCS and 2023 VCS, from 56.7 % to 19.0 % and 12.5 %, respectively. As Lualaba was not one of the 9 initial Mashako Plan Provinces and started MoU implementation in the last quarter of 2021, coverage data is limited, yet, there was a general increase in full immunization coverage between the 2018 MICS (21.1 %), the 2021–22 VCS (38.7 %) and the 2023 VCS (44.2 %).

Beyond immunization coverage numbers, successes in RI implementation were also assessed by a number of Mashako Plan indicator scores (from the monthly mobile supervision data). Under the Mashako Plan, provinces were provided scores for each of the following categories:

• Supervision: percentage of health areas supervised;
• Cold chain: percentage of functional fridges observed;
• Service delivery – fixed: percentage of health areas organizing the minimum level of fixed immunization sessions dictated by national standards;
• Service delivery – outreach: percentage of health areas organizing the minimum level of outreach immunization sessions dictated by national standards;
• Vaccine availability: percentage of health areas with minimum stocks of 80 % antigens and consumables required for full immunization;
• Follow-up of children to minimize vaccine dropouts through organizing household visits;
• An aggregate score that was a weighted average of all indicators listed above.

**Fig. 4:**
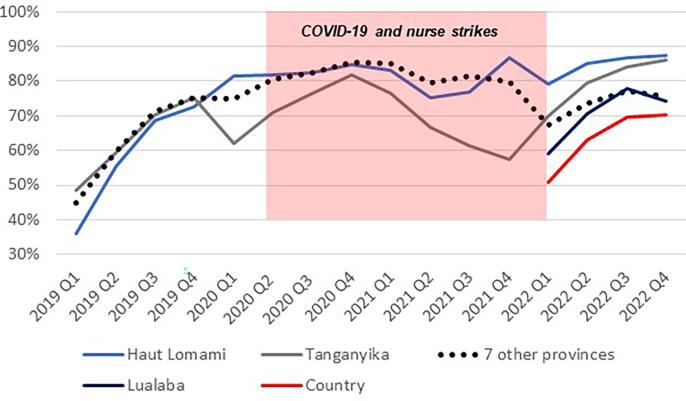
Mashako Plan aggregate score for all indicators over MoU implementation time.

**Fig. 5:**
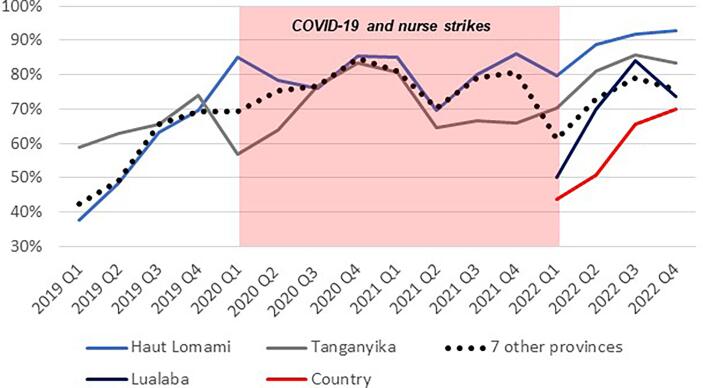
Mashako Plan Vaccine availability score over MoU implementation time.

**Fig. 6:**
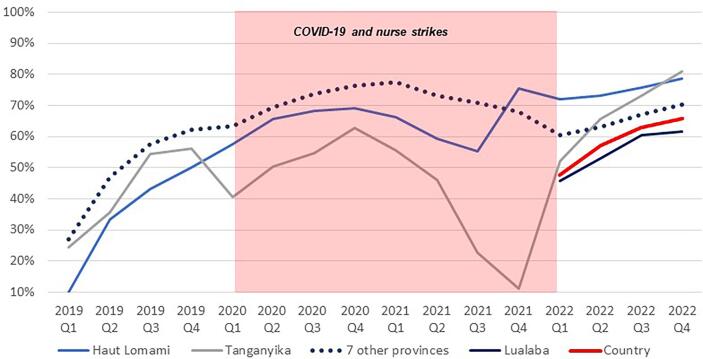
Mashako plan Immunization sessions availability score over MoU implementation time (add in a note that in early 2022, the indicator was divided into fixed and outreach availability, and from there, the average of both indicators is showed here).

Following the implementation of the MoUs, the Mashako Plan aggregate scores increased in all three provinces (Fig. 4). The fluctuation through 2020 and into 2021 is attributed to impact of the COVID-19 pandemic and other barriers to sustained immunization service delivery, including the HCW strikes that caused health facility closures in 2021. Since early 2022, Tanganyika and Haut Lomami have outperformed other provinces in the implementation of Mashako Plan interventions. Similar trends were observed for vaccine availability (Fig. 5**)** and immunization sessions (Fig. 6); in Haut Lomami, a sudden increase throughout 2019, and indication that the COVID-19 pandemic and HCW strikes had limited impact on the availability of vaccines. Yet in Tanganyika, the HCW strike had a greater impact, reducing the vaccination sessions score to 10 %.

### 3.2 MoU Objective 2 – Minimize Polio Circulation and Transmission of VDPV cases and MoU Objective 3 - increase vaccine equity and reduce morbidity and mortality

In addition to increasing and sustaining childhood immunization coverage, the MoU agreements also included an objective to stop the spread of VDPVs and keep provinces polio-free. The number of confirmed cVDPV2 cases in both Haut Lomami and Tanganyika dropped significantly after the implementation of the MoUs, and in both 2020 and 2021, no cases were recorded (Table 3**)**. However, outbreaks of confirmed cVDPV2 in 2022 surged countrywide – especially in Tanganyika and Haut Lomami provinces. This is likely linked to the cVDPV2 emergence in Maniema Province, the province with the second highest number of zero-dose children in the country and further expansion across provincial borders to Tanganyika and Haut-Lomami. Additionally in 2022, there was the emergence of cVDPV1 in Tanganyika (22 cases), which may be attributed to a drop in vaccine coverage in 2021, and eventually spread to Haut Lomami (104) and other provinces (18 cases). As of September 20, 2023, Tanganyika and Haut Lomami had confirmed 21 and 7 cases, respectively, of VDPV1 for 2023. This highlights the continued vulnerability of the immunization system both under the national EPI and MoU agreements in Haut Lomami and Tanganyika. As of 2022, there are no updated data available regarding mortality estimates for the MoU provinces. Additional support to vulnerable populations and improvements of the system are required to prevent future outbreaks.

**Table 3:** Number of confirmed VDPV2 (cVDPV and aVDPV) cases by year, 2017–2022.

	2017	2018	2019	2020	2021	2022
Haut Lomami	8	2	19	0	0	82
Tanganyika	14	3	1	0	0	141
Lualaba	0	0	0	0	0	11
Total 9 Initial Mashako Plan Provinces	22	21	47	16	4	262
Total DRC	24	22	92	86	28	375

### 3.3 MoU Objective 4 – provincial ownership of immunization delivery

Provincial ownership was assessed under three primary targets:

1. Financial autonomy

**Fig. 7:**
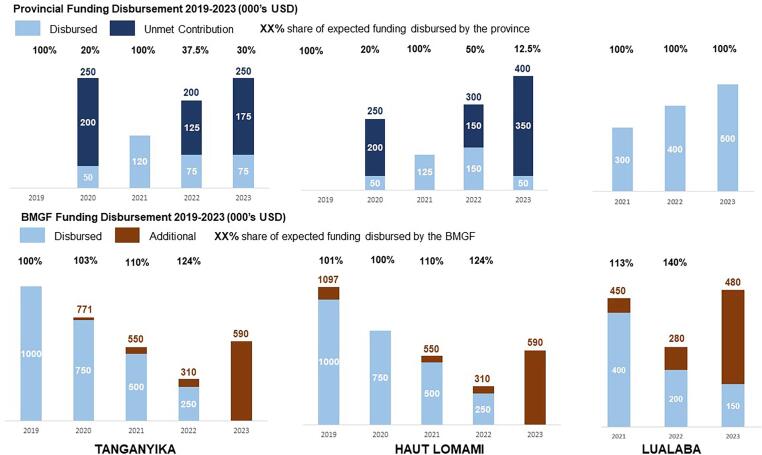
Expected and Actual Immunization Funding Disbursement in all three MoU Provinces 2019–2023. Note: 2023 figures are current as of October 2023, and may have further adjustments by the end of the year. In July, BMGF increased their support to the provinces to focus on "zero dose" activities.

Disbursement data has shown the provinces increased contributions to the basket funds over time, though less than originally expected. In both 2020 and 2021, Haut Lomami and Tanganyika were partly successful in meeting funding targets for RI. Given the difficulties for the provinces to reach the original set targets, new targets were renegotiated in 2021. In both Haut Lomami and Tanganyika the expected percentage share (Fig. 7 **and** Table 1) of funds has not yet been reached. However, the increasing contribution and continued provincial government implication to improve RI indicators is a step towards financial independence. Additionally, friendly competition between the MoU provinces has helped motivate more timely payment into the basket funds.

Haut Lomami was successful in implementing and using financial and accounting software for the MoU, whereas Tanganyika has yet to complete this step. The Haut-Lomami DPS is now using software to account for its health spending and financial contributions from other donors. Regarding financial accountability to external donors, as of July 2022, one external audit had been completed in Haut Lomami and Tanganyika. Based on criteria of agency representation and prior experience, Deloitte was selected by BMGF and provincial leadership to conduct external audits of the MoU implementation from 2019 to 2021. The Deloitte audit for the Haut-Lomami accounts from 2019 to 2021 showed adequate management of MoU funds by the province with no major exceptions and recommended minor improvements for archiving and compliance with procedures. The results from the Tanganyika audit are still pending.

2. Provincial empowerment

Implementation of the MoUs in Haut Lomami and Tanganyika was successful in bolstering provincial ownership and management of RI. As per mobile supervision data, the provinces adhered to the meeting schedules outlined in the MoU. These were essential to increased accountability and responsibility for routine immunization in the provinces. Each year, the governors were expected to host at least two meetings. Haut Lomami and Lualaba both met this target each year. Tanganyika has held at least one meeting per year, but has had a number of political disruptions since 2019 due to frequent changes in governors.

3. Technical assistance

Thus far, technical assistance and external funding of RI have been reduced in both Haut Lomami and Tanganyika. VillageReach concluded its support to logistics by 2021 and UCLA finalized its support for evaluations in 2022. Before the end of 2023, the PATH EPI advisors will conclude their support. However, financial management support will continue for at least 2 additional years. In addition, the McKing consultants—hired by BMGF—will continue their overall support for the implementation of the MoU. While the technical assistance consortium is still active in its advisory role, most aspects of the routine immunization system are now directed and administered by provincial authorities. Financial management and governance still require continued support.

## 4 Conclusions and lessons learned

Ultimately, the implementation of provincial MoUs were successful in increasing subnational involvement in RI and creating vested interest among provincial leadership in immunization coverage. It is important to note that these provinces also benefited from the financial and technical support for routine immunization and health systems strengthening from partners including USAID, World Bank, Gavi, the Vaccine Alliance, UNICEF and WHO.

Several MoU-driven interventions were successful: HCWs were trained, activities were paid via mobile money and bank transfers under the zero-cash policy, monthly monitoring was conducted by EPI supervision, and health zones increased the frequency and regularity of immunization sessions and were able to improve availability of vaccine antigens. Cold chain availability and functionality increased in all health zones of MoU provinces.

Despite success, the MoU process has faced and continues to face, a multitude of challenges. For example, Tanganyika has struggled to implement activities and improve key indicators during the first three years of the program due to several changes in leadership at the technical and political level. The province was affected by HCW strikes for almost half a year—without a health care workforce, standard health services like RI were not delivered in 2021. Additionally, the worldwide COVID-19 pandemic also created disruptions both in service availability (global supply chain) and demand throughout the country, including in the MoU provinces. Furthermore, political interference led to impunity during inspections. External audits were delayed and instead organized in 2022 for the 2019–21 period.

From a financial perspective, MoU provinces ultimately increased their monetary contributions, with Lualaba reaching 100 % of its expected contributions to-date. The provinces provided the first template in the DRC of consistent funding by provincial authorities for RI services. Additional legislative successes included securing funding for RI activities in provincial budgets (RI is now an official line item), and a positive perception towards vaccination among provincial authorities. Despite increases, fund disbursement by the provinces required long negotiations between parties and were less than initially expected. Initial contribution targets may have been overly ambitious and had to be adjusted to more realistic goals. Tanganyika and Haut Lomami had four changes of governors and ministers during the implementation of the MoU, which required re-starting advocacy and commitment to immunization funding with each change in leadership. However, despite these changes and re-commitment, the approach of using a core group to help ensure that the MoU objectives were not completely stopped or changed worked– highlighting a resilience of the program. In the future, MoU implementation could be improved through formal integration into longer standing entities with national and provincial representation (such as the REPACAV) to ensure that leadership turnover and eventual decline in technical support do not disrupt improvements to RI and subnational autonomy.

Haut-Lomami, overall, improved its financial management with the support of the MoUs. Average annual budget utilization of MoU funds was 75 % in Haut-Lomami versus 49 % in Tanganyika over the 2019–2022 period. Tanganyika’s core group composition differed from that of Haut Lomami, with less involvement from the provincial ministry of finance and budget, which may account for some of these differences in implementation. Financial challenges included: an initial lack of understanding on how to appropriate funding for RI by the legislative assemblies, reduction of external partner funds, and minor issues with implementation of the zero-cash policy. Issues with zero-cash policy included delays in activities such as the disbursement of funding to HCWs, and anecdotal reports of local HCWs not receiving full payments—especially in Tanganyika. These issues have been slowly resolved over time as mobile money payments become standard practice. As an incremental approach to financial independence, the implementation of basket accounts beyond provincial budgets are not final solutions–RI activities need to be permanently integrated into the health budget. While this approach was an improvement from previous donor processes, MoUs are still public–private partnership contracts.

In terms of vaccine coverage (MoU Objective 1), Haut-Lomami has reached the targets set and had the highest immunization coverage in the country at the time of the last VCS. Tanganyika and Lualaba are still working towards reaching these objectives. In all three MoU provinces, the percentage of zero-dose children dropped sharply over the implementation period. However, continued analysis of MoU impact is needed to best assess long term changes in the RI system. The goal of 80 % immunization coverage in five years may have been too ambitious as an overarching goal for all provinces. The expected outcomes of MoU implementation should have been altered to better suit the particularities of each province. In tailoring unique objectives for each signing province, the MoU would set more realistic RI targets and seek to sustain targets reached. Future MoUs may consider a two-phase approach: the first phase with process indicator targets of implementation and improved financial management, and the second phase with vaccine coverage targets. Timelines should also be extended from five to ten years to ensure institutional changes in health systems are sustained. In 2022, large outbreaks of cVDPV1 and 2 in both Tanganyika and Haut-Lomami showed the continued fragility of the health systems, despite observed improvements.

While previous MoUs in Nigeria had set similar principal objectives, the MoUs of Haut Lomami, Tanganyika and Lualaba were unique in their design to both implement institutional system changes and monitor and evaluate indicators of improvement thanks to their alignment with the Mashako Plan objectives. At the end of 2022, the provinces and BMGF decided to extend the validity of MoUs by one year, until the end of 2023. In June 2023, Gavi and USAID joined BMGF in a new iteration of the MoUs of Haut-Lomami and Lualaba until 2027 with the following components:

• Add new donors to the basket fund to have a unified immunization budget;
• Adapt specific immunization objectives, targets and activities to the context of each province;
• Add Civil Society Organization members to the decision-making bodies of the MoU and require minimum female participation in these bodies;
• Adapt financial contributions to the budgets of the provinces and improve financial management.

Based on the results observed thus far, continued collaboration with the provincial governments is required to ensure overall sustainability of the MoU interventions. In the DRC, the national government has started preliminary conversations with other provinces and donors to expand the initially successful MoU model to other health interventions beyond routine immunization.

This MoU approach demonstrates that there are both sustainable and impactful approaches to improve immunization coverage in low-income countries. Beyond DRC, the MoU model for partnerships has been reproduced in two countries. As MoU implementation becomes more widespread, these partnerships should be adapted to each sub-regional area – an approach that has been successful for implementation and support of the local government to take an active role in the routine immunization system. Additionally, future MoUs should focus on community engagement and political buy-in to ensure sustained improvements and support following the conclusion of the MoU period. The institutional capabilities of the provinces should be carefully evaluated to improve the odds of success. Memorandums of Understanding can serve as the basis for key partnerships in the improvement of RI and health care delivery services both in the DRC as a whole, and other LMICs worldwide.

## Declarations

### Ethical approval

No human subjects research was conducted as a part of the implementation of the Mashako Plan or the Memorandums of Understanding (MoUs). All authorizations were granted by the government of the DRC and the Ministry of Health. The Kinshasa School of Public Health Vaccine Coverage Study (VCS) was approved by the ethics committee at the University of Kinshasa, DRC (IRB Approval ESP/CE/146/2020).

## Funding

This manuscript was funded by The Bill and Melinda Gates Foundation (Grant Number INV035951).

## Declaration of competing interest

The authors declare that they have no known competing financial interests or personal relationships that could have appeared to influence the work reported in this paper.

## Data Availability

Data will be made available on request.
